# Danazol induces apoptosis and cytotoxicity of leukemic cells alone and in combination with purine nucleoside analogs in chronic lymphocytic leukemia

**DOI:** 10.1007/s00277-015-2579-5

**Published:** 2015-12-22

**Authors:** Monika Podhorecka, Arkadiusz Macheta, Sylwia Chocholska, Agnieszka Bojarska-Junak, Agnieszka Szymczyk, Aneta Goracy, Anna Dmoszynska, Marek Hus

**Affiliations:** Department of Haematooncology and Bone Marrow Transplantation, Medical University of Lublin, Staszica 11, 20-081 Lublin, Poland; Department of Clinical Immunology, Medical University of Lublin, Lublin, Poland

**Keywords:** Apoptosis, Bendamustine, Chronic lymphocytic leukemia, Cladribine, Cytotoxicity, Danazol, Fludarabine

## Abstract

Recently, great progress has been achieved in the treatment of chronic lymphocytic leukemia (CLL). However, some patients, particularly older patients with comorbidities or with relapsed/refractory leukemia, still have limited therapeutic options. There is an urgent need to discover less toxic and more effective drugs for CLL patients. Applying new modalities or substances that are widely used for the treatment of other diseases has been reported to improve results in CLL treatment. This study aimed to assess the non-chemotherapeutic drug danazol for its potential to destroy leukemic cells. Leukemic cells, obtained from the peripheral blood and bone marrow of 23 CLL patients, were cultured in the presence of danazol and its combination with the purine nucleoside analogs fludarabine and cladribine and bendamustine. After 24 h of incubation, the rate of apoptosis indicated by active caspase-3 expression, and cytotoxicity indicated by forward light scatter and light scatter analysis, was assessed by flow cytometry. We also measured expression of apoptosis-regulating proteins of BCL family and active caspase 9 and active caspase 8 expressions in leukemic cells. Danazol had a caspase-dependent pro-apoptotic and cytotoxic effect on leukemic cells in a tumor-specific manner. The mechanisms of its action appear to be complex and should be precisely established; however, induction of apoptosis involving both mitochondrial and receptor cascades appears to be most probable. Danazol showed a synergic effect with cladribine, an additive effect with fludarabine, and an infra-additive effect with bendamustine. The rate of danazol-induced apoptosis and cytotoxicity did not differ between patients with better and worse prognostic markers. Our results indicate that danazol may be a potential therapeutic agent for CLL patients alone and in combination with purine analogs.

## Introduction

The pathogenesis of chronic lymphocytic leukemia (CLL) has not been yet adequately established. Nevertheless, abnormal apoptosis of lymphocytes has been recognized to play an important role in the pathogenesis of CLL. Leukemic transformation is initiated by genomic alterations, causing deletion of specific micro-RNA genes and increasing the resistance of B cells towards apoptosis [1, 2]. The role of B cell receptor (BCR) signaling in the pathogenesis of CLL was recently recognized, based on structural restrictions of the BCR and BCR-dependent survival and growth of malignant B cells [3]. The biology of CLL is also directly connected with its microenvironment in which accessory cells can promote cell growth and survival in leukemia. These disorders lead to accumulation of the clonal B cells CD5+/CD19+/CD23+ in the peripheral blood, bone marrow, and lymphoid organs [4].

CLL can be effectively treated with purine analogs, alkylating agents, bendamustine, glucocorticoids, or monoclonal antibodies. During recent years, great progress in targeted therapy of CLL has been made. Novel classes of drugs, such as oral tyrosine kinase inhibitors (ibrutinib, idelalisib) and BCL-2 protein antagonists, which induce apoptosis in leukemic cells, have received approval for the treatment of CLL patients [5, 6]. Despite new therapeutic options, CLL is still an incurable disease. Some patients, especially older patients with comorbidities or relapsed/refractory leukemia, have limited therapeutic options. Therefore, there is an urgent need to discover less toxic and more effective drugs for CLL patients [6, 7]. Applying new modalities or non-chemotherapeutic substances that are widely used in the treatment of other diseases has been reported to improve results in CLL treatment, as shown in our previous studies [8, 9].

Danazol is an attenuated synthetic hormone with weak androgenic effects. Danazol has unique properties similar to corticosteroids and a structure related to testosterone and ethisterone. This drug is widely used in the treatment of endometriosis and mammary gland dysplasia. In hematology, danazol is used with success in immune thrombocytopenic purpura and autoimmune hemolytic anemia [10–15]. The use of danazol therapy in neoplastic hematological proliferations, such as myeloproliferative neoplasms and myelodysplastic syndromes, has also been reported [16–18].

This study aimed to assess the role of danazol as an inducer of apoptosis and cytotoxicity in CLL. Danazol as a single agent and in combination with drugs that are used in CLL therapy (the two purine nucleoside analogs fludarabine and cladribine, and bendamustine) was added to in vitro cultures of leukemic cells. The rate of apoptosis and cytotoxicity induced by these drugs was assessed with the flow cytometry method. The obtained results were then analyzed regarding prognostic factors of CLL.

## Material and methods

### Research material

Twenty-three CLL patients who were diagnosed in the Department of Haematooncology and Bone Marrow Transplantation, Medical University of Lublin, previously not treated, constituted the study group. The diagnosis of CLL was based on clinical examinations and morphological and immunological standards. Agreement for the research was received from the local bioethics committee, and the patients were asked to sign informed consents. Samples of the peripheral blood and bone marrow were collected in syringes with the anticoagulant edetate (Ethical Committee of Medical University of Lublin, Sarstedt, Germany).

### Isolation of cells

Peripheral blood mononuclear cells and bone marrow mononuclear cells were separated by Biocoll (AG Biochrom, Germany) density gradient centrifugation. After washing with phosphate-buffered saline, the number and viability of cells were assessed with trypan blue staining. Viability below 95 % was a disqualifying criterion for further study.

### Cell cultures

Isolated cells at a concentration of 2 × 10^6^/ml were resuspended in a liquid culture medium consisting of RPMI 1640 with L-glutamine, penicillin, streptomycin, and 10 % fetal calf serum. This culture medium was supplemented with 10 μM danazol, 1 μg/ml fludarabine, 1. 4 μg/ml cladribine, 20 μM bendamustine, or a mixture of danazol and one of these drugs. We selected a danazol concentration of 10 μM based on preliminary experiments in CLL performed by Tung et al. [19]. To obtain stock solutions, danazol was dissolved in ethanol. We selected the concentrations of other drugs based on the literature and our preliminary experiments in CLL cultures, in which certain concentrations caused a significant induction in apoptosis with spontaneous apoptosis that did not exceed 50 % of the total cell culture. All of the reagents were obtained from Sigma-Aldrich Chemie GmbH (Germany). The cells were cultured at 37 °C in a 5 % CO_2_ atmosphere. Cells were exposed to the drugs for 0 and 24 h in culture, and cells that were treated for 24 h were assessed for the frequency of apoptosis and cytotoxicity. Cell samples that were incubated in the absence of any drug and in the presence of a drug solvent for periods of time that were equivalent to the drug-treated cells were considered negative controls. After the required period of incubation, the cells were then subjected to the procedures described below.

### Measurement of apoptosis by detection of active caspase-3 expression

Cells that had been treated in culture with drugs were initially incubated for 15 min with anti-CD19 PE-Cy7 and anti-CD5 APC-conjugated monoclonal antibodies (mAbs) (Pharmingen, USA) at room temperature. Subsequently, the cells were subjected to fixation and permeabilization procedures using the BD Cytofix/Cytoperm kit (BD Biosciences, USA) according to the manufacturer’s instructions. The cells were then incubated with FITC-conjugated anti-active caspase-3 mAb (Pharmingen, USA). Cells that were incubated with Z-VAD-FMK were treated as a negative control.

Acquisition and analysis of the data were carried out by flow cytometry method using the eight-color flow cytometer FACSCanto II with FACSDiva Software (Becton Dickinson, USA). At least 10,000 cells of each collected sample were analyzed and populations of leukemic cells (CD5+/CD19+) were separated. Further experiments were carried out within this population. Apoptosis was evaluated by assessment of the percentage of cells expressing active caspase-3. The assay of non-leukemic cells was performed in the CD19+/CD5− population, which represents normal B lymphocytes.

### Measurement of cytotoxicity using forward light scatter and side light scatter analysis

The danazol-treated cells that were gated according to CD19 and CD5 expression (see the previous section) were also analyzed by flow cytometer by forward light scatter (FSC) and side light scatter (SSC) parameters. Changes in light scattering properties of cells occur during apoptosis. Late apoptotic/secondary necrotic cells are characterized by markedly diminished scatter parameters [20, 21]. At least 10,000 cells of each sample were collected, and the populations of the leukemic cells CD5+/CD19+ and non-leukemic B cells CD19+/CD5− were analyzed using FSC and SSC parameters.

The experiments with caspase inhibitor Z-VAD-FMK were done to assess non-specific binding of anti-active caspase 3 mAb as negative control (mentioned above) and whether cytotoxic activity of danazol is blocked by caspase inhibitors. Z-VAD-FMK was added to peripheral blood and bone marrow cell cultures with danazol, and then, cytotoxic effect was analyzed by measuring FSC and SSC.

### Assessment of active caspase-8, active caspase-9, BCL-2, BAX, and BCL-XL protein expression in leukemic cells

Peripheral blood and bone marrow cells that had been treated in culture with danazol were incubated for 15 min with anti-CD19, PE-Cy7, and anti-CD5 APC-conjugated mAbs (see previous section). The cells were then subjected to fixation and permeabilization procedures using the BD Cytofix/Cytoperm kit (BD Biosciences) according to the manufacturer’s instructions. The cells were then incubated with PE-conjugated anti-intracellular BAX mAb, PE-conjugated anti-intracellular BCL-2 mAb, PE-conjugated anti-intracellular BCL-XL (Santa Cruz Biotechnology, USA), or an isotype-matched negative control (DAKO, Denmark) in the dark at room temperature for 15 min.

We used an Abcam kit (USA) to assess expression of active caspase-8 and active caspase-9. The cells indicated for CD19 and CD5 expression (see above) were incubated with FITC-IETD-FMK and FITC-LEHD-FMK, respectively, according to the manufacturer’s instructions. Cells that were incubated with Z-VAD-FMK were treated as a negative control.

Acquisition and analysis of the data were carried out by flow cytometry within the population of leukemic cells (CD5+/CD19+). The percentage of cells expressing active caspase-8 and active caspase-9 and mean fluorescence intensity of BCL-XL, BCL-2, and BAX were analyzed. The ratio of BCL-2/BAX mean fluorescence intensity was calculated.

### Calculation of the cooperative index

To assess the type of interaction between danazol and fludarabine, cladribine, or bendamustine, the cooperative index (CI), which is a parameter based on the Chou–Talalay method, was estimated [22]. The following formula was used: CI = sum of specific apoptosis induced by treatment with a single drug/specific apoptosis defined with combined agents.

The percentage of specific apoptosis was determined using the following formula: specific apoptosis = (drug − induced apoptosis − spontaneous apoptosis)/(100 − spontaneous apoptosis) × 100 %

The results were characterized as synergic, addictive, and infra-additive, when CI < 1, CI = 1, and CI > 1, respectively.

### Analysis of ZAP-70 and CD38 expression

A total of approximately 1 × 10^6^ peripheral blood cells were stained with the mAbs CD19 PE-Cy7, CD5 APC (Pharmingen, USA) or CD3 PE (BD Pharmingen). Following membrane staining, the cells were fixed by 1 % paraformaldehyde solution in phosphate-buffered saline for 15 min at room temperature and permeabilized with 70 % ethanol for 1 h at −20 °C. After washing, anti-ZAP-70 antibody (Biomol Research Laboratories, USA) that was labeled by the ZenonTM Alexa Fluor® 488 Mouse IgG2a Labeling Kit (Molecular Probes, USA) was added to the sample tubes. The samples were incubated for 30 min, washed, and examined by flow cytometry method. Patients were considered positive for ZAP-70 when its expression was detected in ≥20 % of leukemic cells. To assess CD38 expression, peripheral blood mononuclear cells were stained with anti-CD38 FITC (BD Pharmingen), anti-CD19 PE, anti-CD5 CyChrome mAbs, or IgG1 isotypic control for 20 min in darkness, and they were analyzed with flow cytometry method. Patients were considered CD38 positive when expression was found in at least 20 % of CLL cells.

### Fluorescence in situ hybridization

Fluorescence in situ hybridization was used to determine leukemic cells with cytogenetic abnormalities that are relevant markers of prognosis of CLL. This procedure was performed according to the manufacturer’s instructions. The locus-specific probes 17p13.1 (LSI TP53), 11q22.3 (LSI ATM), 13q14.3 (D13S319), 13q34, and the chromosome 12 centromere (Abott Diagnostics, USA) were used. Probes were denatured at 73 °C for 5 min and then applied to the designated areas of the slides. Following an overnight hybridization at 37 °C, the slides were washed and air-dried in the dark. The slides were then stained with DAPI and stored at −20 °C in the dark. The samples were analyzed using a BX51 fluorescence microscope (Olympus, USA), and images were captured with the CytoVision image analysis system. At least 200 nuclei were assessed for each probe, and the cutoff value for each probe was 20 %.

### Statistical analysis

Statistical analysis was performed using STATISTICA 8.0 software for Windows. All results are shown as mean values with standard deviation or median. The Mann–Whitney *U* test was used to evaluate the differences between subgroups of patients. A value of *p* < 0.05 was considered to be statistically significant.

## Results

### Danazol induces apoptosis and cytotoxicity of leukemic cells in cell cultures in a tumor-specific manner

**Fig. 1:**
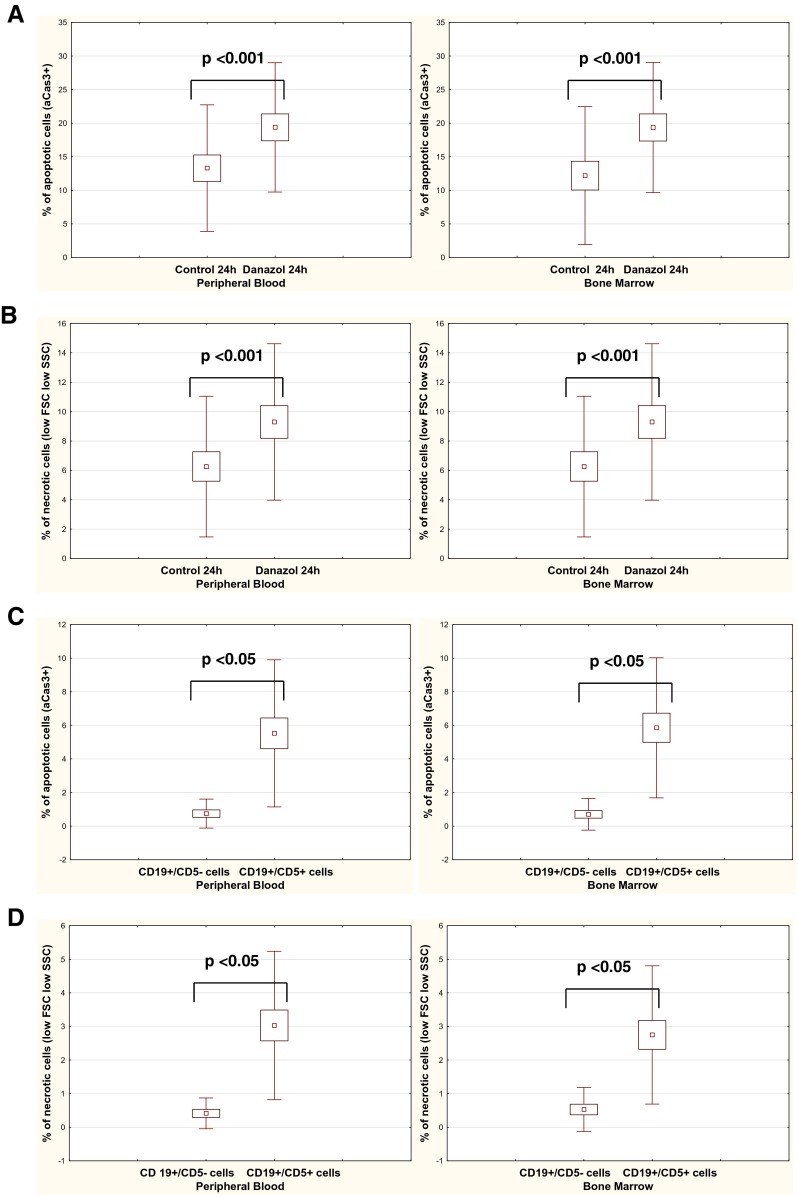
Percentage of apoptotic leukemic (CD19+/CD5+) cells with active caspase-3 expression (aCas3+) after 24 h of culture with danazol in peripheral blood and bone marrow samples compared with control cultures (**a**). Percentage of necrotic leukemic (CD19+/CD5+) cells with low forward light scatter (low FSC) and low side light scatter (low SSC) after 24 h of culture with danazol in peripheral blood and bone marrow samples compared with control cultures (**b**). Percentage of apoptotic cells within a population of non-leukemic B cells (CD19+/CD5−) compared with a population of leukemic cells (19+/CD5+). Data show the danazol-induced increase in the percentage of apoptotic cells above the values observed in control cultures (**c**). Percentage of necrotic cells within a population of non-leukemic B cells (CD19+/CD5−) compared with a population of leukemic cells (19+/CD5+). Data show the danazol-induced increase in the percentage of apoptotic cells above the values observed in control cultures (**d**). All graphs show the mean ± standard deviation of results obtained from the group of analyzed patients (*n* = 23). The *p* values are indicated

Clinical data from the 23 analyzed patients are shown in Table 1. Figure 1a shows the pro-apoptotic effect of danazol on CLL cells derived from the peripheral blood and bone marrow treated in in vitro environment. Active caspase-3 expression in cultures with danazol was significantly higher in 24-h cultures compared with that in cultures without danazol (control). This increased frequency of danazol-induced apoptosis was observed in peripheral blood and in bone marrow cultures. Figure 1b shows a cytotoxic effect of danazol on leukemic cells in the peripheral blood and bone marrow that was assessed by distinguishing the population of cells with diminished FSC and SSC parameters. The percentage of necrotic cells was significantly higher after 24 h of incubation in danazol-induced cultures compared with control cultures in peripheral blood and bone marrow samples. These results suggest that danazol is an inductor of apoptosis and cytotoxicity in leukemic cells. The experiments with caspase inhibitor Z-VAD-FMK performed in peripheral blood and bone marrow samples showed that percentage of necrotic cells was significantly lower in 24-h cultures with danazol and caspase inhibitor in comparison to the cultures without inhibitor (*p* < 0.001). Thus, it can be concluded that the cytotoxic effect of danazol is caused by caspase-dependent apoptosis. There was a significant correlation between the rate of apoptosis and the rate of cytotoxicity. Therefore, in further analysis, we only presented the rate of apoptosis for clarification.

**Table 1:** Clinical characteristics of patients with chronic lymphocytic leukemia who were enrolled in the study

Number of patients	23
Sex	
Male	12
Female	11
Age	44-83
Median	69
Rai stage	
0	6
1	4
2	9
3	2
4	2
CD38 (cutoff 20 %)	
Positive	8
Negative	14
Not available	1
ZAP-70 (cutoff 20 %)	
Positive	6
Negative	16
Not available	1
Cytogenetics	
Low-risk or absence of changes	13
High-risk changes	7
Not available	3

To determine the effects of danazol on normal B cells, we assessed the percentage of apoptotic and necrotic cells within a population of CD19+/CD5− cells derived from CLL patients. Analysis was performed in 17 out of 23 patients’ peripheral blood samples and in all patients’ bone marrow samples where the percentage of normal B cells was higher than 1 % (Fig. 1c, d). There was no significant apoptotic and cytotoxic effects in this group of cells. A significantly lower percentage of apoptotic and necrotic cells was observed in the population of non-leukemic cells than in the population of leukemic cells in peripheral blood and bone marrow samples.

### Danazol affects the expression of apoptosis-regulating proteins in leukemic cells

We found statistically significant differences in some apoptosis-regulating proteins between danazol-induced cultures and control cultures. There was a significant decrease in the BCL-2/BAX ratio in CLL cells after 24 h of cell culture with danazol compared with that at 0 and 24 h of control culture. Intracellular expression of BCL-XL protein was also significantly lower in danazol cultures after 24 h of incubation than in control 0- and 24-h cultures. The results are presented in Table 2. These results support the pro-apoptotic effect of danazol on leukemic cells.

**Table 2:** Expression of apoptosis-regulating proteins in leukemic cells at 24 h of culture with danazol and in control culture

	Control 24 h	Danazol 24 h	*p*	Control 24 h	Danazol 24 h	*p*
	Peripheral blood			Bone marrow		
BCL-2/BAX ratio	0.54 ± 0.31	0.26 ± 0.19	0.001	0.46 ± 0.24	0.25 ± 0.21	p < 0.001
BCL-XL (MFI)	407.34 ± 216	330.6 ± 154.6	0.001	407.3 ± 216.04	330.66 ± 152.4	p < 0.001
aCas8 (%)	26.27 ± 7.1	30.5 ± 5.5	0.001	23.1 ± 5.8	27.8 ± 5.4	p < 0.001
aCas9 (%)	24.3 ± 4.6	26.8 ± 3.3	0.05	21.7 ± 4.6	25.7 ± 3.9	p < 0.05
	Peripheral blood			Bone marrow		
aCas8 (%) above the level of control culture	4.37 ± 4.25			5.41 ± 3.6		
aCas9 (%) above the level of control culture	2.97 ± 2.98			4.2 ± 4.1		
*p*	NS			NS		

### Danazol induces the caspase-8 and caspase-9 apoptotic cascade in leukemic cells

To determine which apoptotic cascade is activated by danazol, we assessed expression of the main caspases that are involved in mitochondrial and receptor-mediated apoptosis (i.e., active caspase-9 and active caspase-8, respectively). We observed a significant increase in percentage of CLL cells with active caspase 9 and active caspase 8 expressions after 24 h of cell culture with danazol compared with that at 24 h of control culture. Interestingly, the percentage of CLL cells in 24-h cultures with danazol was not different regarding expression of active caspase-9 and active caspase-8 (Table 2). This finding suggests that danazol is able to induce both the mitochondrial and receptor pathways of apoptosis.

### Danazol and fludarabine have an additive effect on apoptosis of leukemic cells

**Fig. 2:**
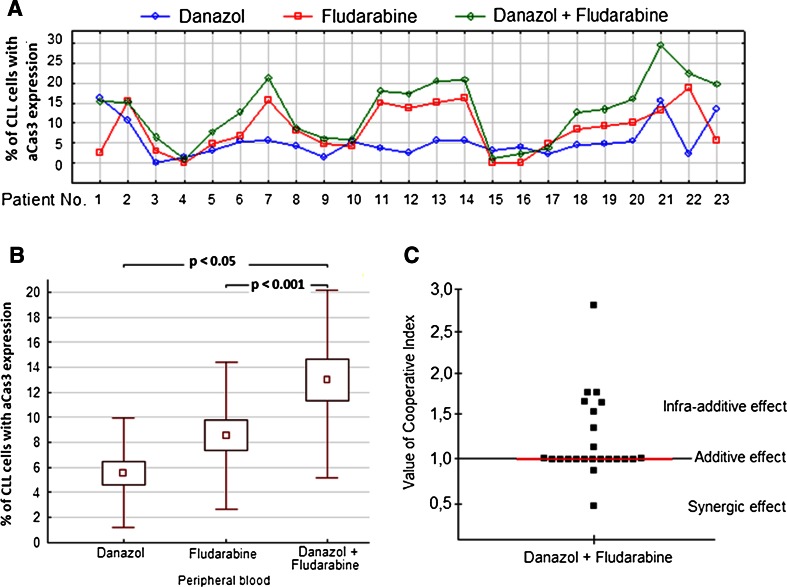
Percentage of leukemic cells with expression of active caspase-3 (aCas3) in 24-h cultures with danazol (10 μM), fludarabine (1 μg/ml), and both drugs (results of individual cases) (**a**). Results shown as mean ± standard deviation (**b**). The value of the CI calculated for each probe. The *red line* represents the median value (**c**). Data represent the drug-induced increase in the percentage of apoptotic cells above values observed in parallel control cultures without drugs

To assess the rate of apoptosis induced by danazol in combination with fludarabine, we measured the expression of active caspase-3 in a population of CD19+/CD5+ cells derived from peripheral blood in cultures with danazol and fludarabine. Figure 2 shows the drug-induced increase in frequency of apoptotic cells above the level of spontaneous apoptosis seen in the 24-h parallel control cultures without drug. As evident in Fig. 2, the frequency of apoptosis in danazol and fludarabine-treated cultures was significantly higher than that in danazol-treated cultures and the same was true when compared with fludarabine-treated cultures. To evaluate the effect of the combined action of danazol and fludarabine, the CI for each sample was calculated. The CI for danazol and fludarabine was 1 for 13 out of 23 analyzed patients, lower than 1 for two samples, and higher than 1 for eight patients (Fig. 2b). Because the median CI value was 1, an additive effect of these drugs in combination was presumed (Fig. 2c).

### Danazol synergizes with cladribine to induce apoptosis of leukemic cells

**Fig. 3:**
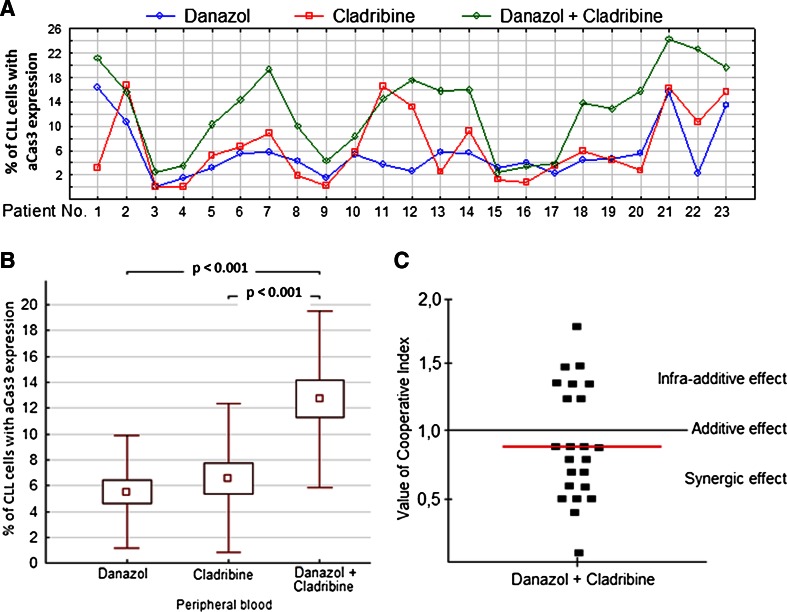
Percentage of leukemic cells with expression of active caspase-3 (aCas3) in 24-h cultures with danazol (10 μM), cladribine (1.4 μg/ml), and both drugs (results of individual cases) (**a**). Results shown as mean ± standard deviation (**b**). The value of the CI calculated for each probe. The *red line* represents the median value (**c**). Data represent the drug-induced increase in the percentage of apoptotic cells above values observed in parallel control cultures without drugs

To evaluate the rate of apoptosis caused by danazol and cladribine, we analyzed the expression of active caspase-3 in a peripheral blood leukemic cell population in cultures with danazol and cladribine. The rate of apoptosis was significantly higher in cultures treated with both danazol and cladribine compared with danazol cultures and cladribine cultures (Fig. 3a, b). The CI calculated for this assay was lower than 1 in most patients (15/23), with a median value of 1. These results suggested a synergic effect of the interaction (Fig. 3c).

### Danazol and bendamustine have an infra-additive effect on induction of apoptosis of leukemic cells

**Fig. 4:**
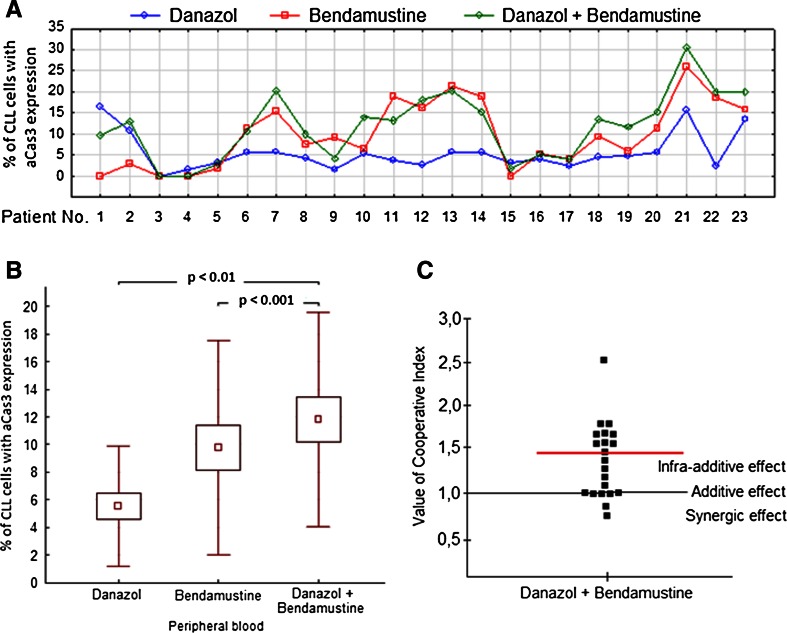
Percentage of leukemic cells with expression of active caspase-3 (aCas3) in 24-h cultures with danazol (10 μM), bendamustine (20 μM), and both drugs (results of individual cases) (**a**). Results shown as mean ± standard deviation (**b**). The value of the CI calculated for each probe. The *red line* represents the median value (**c**). Data represent the drug-induced increase in the percentage of apoptotic cells above values observed in parallel control cultures without drugs

We further evaluated the combined effects of danazol and bendamustine. We found that the frequency of apoptosis caused by danazol in combination with bendamustine was significantly higher than that observed in single drug-treated cultures (Fig. 4a, b). However, the CI for 14 out of 23 patients was higher than 1, with a median value higher than 1. Therefore, these results suggested an infra-additive effect of these drugs in combination (Fig. 4c).

### Rate of danazol-induced apoptosis of leukemic cells is independent of prognostic factors

**Fig. 5:**
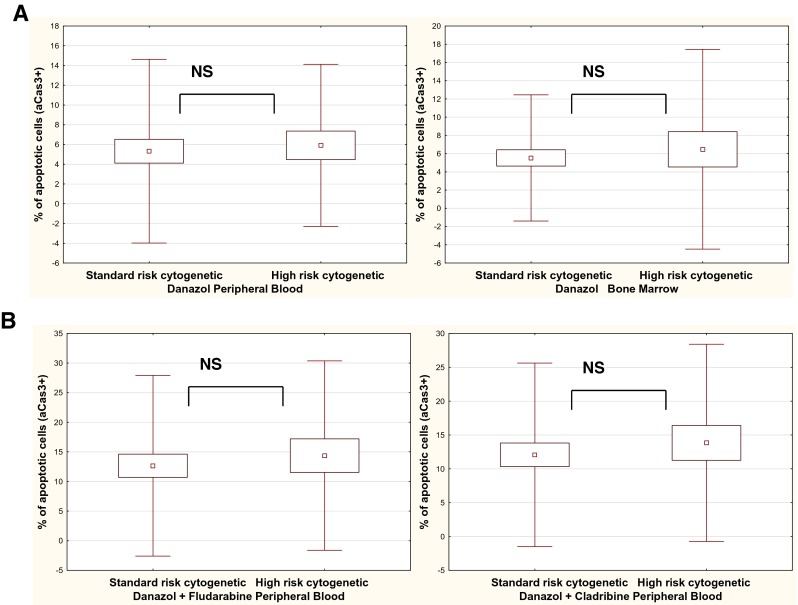
Percentage of apoptotic leukemic cells with active caspase-3 expression (aCas3) in 24-h culture with danazol in peripheral blood and bone marrow samples in the standard-risk cytogenetic group (del13q14.3, trisomy 12, or no changes detected) and in the high-risk cytogenetic group (17p13.1 or 11q22.3) (**a**). Percentage of apoptotic leukemic cells with aCas3+ in 24-h cultures with danazol and fludarabine or danazol and cladribine in peripheral blood samples in the standard-risk cytogenetic group and high-risk cytogenetic group (**B**). Data represent the drug-induced increase in the percentage of apoptotic cells above values observed in parallel control cultures without drugs. All graphs show mean ± standard deviation. *NS* not statistically significant

The results of danazol-induced apoptosis were compared between patients with standard prognostic markers and those with a worse prognosis. There were no significant differences in rate of apoptosis between these two groups in cultures with danazol or in cultures supplemented with danazol and other drugs as far as clinical stadium of the disease and ZAP-70 and CD38 expression were concerned. Interestingly, there was no difference as far as the presence of cytogenetic changes was concerned, either. The rate of apoptosis induced by danazol was comparable in the group of patients with del17p13.1 or del11q22.3 and in the group with 13q14.3 deletion or trisomy 12 (Fig. 5). These findings suggest that the pro-apoptotic effect of danazol is independent of prognostic factors in CLL patients and this drug may sensitize high-risk patients.

## Discussion

Despite major progress in the treatment of CLL in recent years, this disease is still incurable. A lot of research has focused on searching for the optimal therapeutic regimens to achieve therapeutic goals in CLL, which in most patients are to acquire complete remission and long overall survival. The best results in this field are currently achieved with chemotherapy based on the purine nucleoside analogs in combination with alkylating agents and monoclonal antibodies. The use of such a regimen is still the gold standard for first-line treatment of fit patients with CLL, despite new forms of targeted treatment, such as oral tyrosine kinase inhibitors and BCL-2 protein antagonists. However, such therapy is limited in elderly patients with a poor performance status or severe comorbidities. Moreover, in some patients with limited disease, starting cytostatic treatment is not necessary until disease progression [23, 24]. Therefore, it is of importance to investigate new, more effective and less toxic drugs for CLL patients, which could be used just to control disease when there is no need to start chemotherapy or to reduce doses of chemotherapeutic agents if treatment should be started. Our study aimed to assess whether danazol could play such a role.

Danazol has successfully been used in some hematological diseases such as immune thrombocytopenic purpura and autoimmune hemolytic anemia [10–15]. There are also reports in the form of individual clinical cases indicating that danazol may be a safe and effective alternative for the treatment of advanced myeloproliferative neoplasms, such as myelofibrosis [16, 17]. The use of danazol in therapy of myelodysplastic syndromes is also a possibility [18]. However, the role and the mechanism of action of danazol in these cases are unclear. Danazol-induced apoptosis of CLL cells was first described by Tung et al. [19]. The authors studied patients receiving danazol in combination with glucocorticoids and showed a significant decrease in the leukocyte count, which increased after withdrawal of this drug. Based on these observations, pre-clinical laboratory tests with CLL cells were performed, and these cells showed toxicity of danazol against CD19+/CD5+ cells at concentrations above 3 μM, and the optimal concentration was 10 μM [19]. The mechanism of danazol activity in CLL is unknown. Tung et al. [19] proposed that a reduction in blood CD23 level by danazol observed during endometriosis treatment suggested a direct effect on activated B cells in CLL [25, 26]. Similarly, danazol can decrease production of inflammatory cytokines, such as interleukin-6 and tumor necrosis factor-alpha, which probably can restrict their ability to induce proliferation of CLL cells in vivo [10, 27].

Based on these properties of danazol, we assessed its effects on leukemic cells derived from CLL patients. Consistently with the observations of Tung et al. [19], we showed that danazol-induced caspase-dependent apoptosis and cytotoxicity of CLL cells in the peripheral blood and bone marrow. Danazol-induced apoptosis appears to involve both mitochondrial and membrane cascades because of similar levels of active caspase-9 and active caspase-8 expressions detected in leukemic cells. This process, mainly in part connected with the mitochondrial cascade, involved apoptosis-regulating proteins, including BCL-2, BAX, and BCL-XL. We observed a decrease in the BCL-2/BAX ratio and BCL-XL expression in leukemic cells treated by danazol. To the best of our knowledge, this is the first report of such a mechanism of danazol action in CLL cells. However, literature data regarding diseases other than CLL show a similar mechanism of action of danazol. Tanaka et al. [28] showed that danazol enhanced Fas-mediated apoptosis in human endometrial epithelial cells, indicating involvement of a receptor cascade. However, another study reported that expression of BCL-2 was decreased in adenomyotic endometrium treated with danazol, which indicated activation of a mitochondrial cascade [29]. Additionally, our study indicated that danazol-induced cytotoxicity against leukemic cells occurs in a tumor-specific manner without affecting normal B cells. Such a specific effect may be due to the different features of leukemic and normal cells, such as anti-apoptotic protein expression (BCL-2 family) and membrane receptor expression (e.g., FAS TRAIL), which are changed in leukemic lymphocytes [30].

Our study is the first to analyze the combined effect of danazol and purine analogs or bendamustine on the rate of apoptosis of CLL cells. Danazol showed a synergic effect with cladribine, an additive effect with fludarabine, and an infra-additive effect with bendamustine. Purine nucleotide analogs and bendamustine are treatment options used in CLL patients and higher remission rates can be obtained when they are used. However, some patients are refractory to these drugs. Other problems of these drugs are associated with their toxicity. Immunosuppression with a decrease in the CD4+/CD8+ ratio can occur, which leads to development of opportunistic infections, myelosuppression, and gastrointestinal toxicities, including nausea, vomiting, and hepatic lesions [31–33]. Therefore, a reduction in cytostatic toxicity achieved with a lower dose may be important. Because of the combined effects of danazol with fludarabine and cladribine, lower purine analog doses in combination with danazol are possible.

Because of the heterogeneous clinical course of CLL, prognostic factors of this disease have been defined to predict clinical outcome. They include classic prognostic factors, such as the stage of disease, and new biological factors, such as the gene mutation status of the variable part of immunoglobulin heavy chain protein expression of ZAP-70 and CD38 antigen, and cytogenetic abnormalities [34–36]. In this study, we investigated whether the rate of apoptosis caused by danazol is different in patients with a low-risk and high-risk prognosis. We found that the level of apoptosis was independent of some prognostic factors, including the clinical stage according to Rai and ZAP-70 and CD38 expression. Interestingly, it was also independent of the presence of cytogenetic changes in leukemic cells. Therefore, the use of danazol may be equally effective in both groups of patients with a worse and better prognosis and danazol can sensitize high-risk patients. These findings are similar to those presented by Tung et al. [19], showing efficacy of danazol in patients with unfavorable cytogenetic abnormalities.

## Conclusions

In summary, our results indicate that danazol induces apoptosis and cytotoxicity of CLL cells as a single agent and in combination with purine analogs. The mechanisms of action of danazol appear to be complex and remain to be precisely established. However, the induction of apoptosis involving both mitochondrial and receptor cascades appears to be most probable. Moreover, danazol’s action is independent of the presence of poor prognostic factors. Therefore, danazol could be considered as a therapeutic agent for CLL patients.

## References

[1] Calligaris-Cappio F, Hamblin TJ (1999). B-cell chronic lymphocytic leukemia: a bird of a different feather. J Clin Oncol.

[2] Calin GA, Dumitru CD, Shimizu M (2002). Frequent deletions and downregulation of micro-RNA genes miR15 and miR16 at 13q14 in chronic lymphocytic leukemia. Proc Natl Acad Sci U S A.

[3] Hamblin TJ, Oscier DG (1997). Chronic lymphocytic leukemia: the nature of the leukemic cells. Blood Rev.

[4] Hallek M, Cheson BD, Catovsky D (2008). Guidelines for the diagnosis and treatment of chronic lymphocytic leukemia: a report from the International Workshop on Chronic Lymphocytic Leukemia updating the National Cancer Institute-Working Group 1996 guidelines. Blood.

[5] Tausch E, Mertens D, Stilgenbauer S (2014). Advances in treating chronic lymphocytic leukemia. F1000Prime Rep.

[6] Byrd JC, Jones JJ, Woyach JA, Johnson AJ, Flynn JM (2014). Entering the era of targeted for chronic lymphocytic leukemia: impact on the practicing clinician. J Clin Oncol.

[7] Cuneo A, Cavazzini F, Ciccone M (2014). Modern treatment in chronic lymphocytic leukemia: impact on survival and efficacy in high-risk subgroups. Cancer Med.

[8] Podhorecka M, Halicka D, Klimek P, Kowal M, Chocholska S, Dmoszyńska A (2010). Simvastatin and purine analogs have a synergic effect on apoptosis of chronic lymphocytic leukemia cells. Ann Hematol.

[9] Podhorecka M, Halicka D, Klimek P, Kowal M, Chocholska S, Dmoszyńska A (2011). Resveratrol increases rate of apoptosis caused by purine analogues in malignant lymphocytes of chronic lymphocytic leukemia. Ann Hematol.

[10] Ahn YS, Harrington WJ, Simon SR, Mylvaganam R, Pall LM, So AG (1983). Danazol for the treatment of idiopathic thrombocytopenic purpura. N Engl J Med.

[11] Maloisel F, Andres E, Zimmer J, Noel E, Zamfir A, Koumarianou A (2004). Danazol therapy in patients with chronic idiopathic thrombocytopenic purpura: long-term results. Am J Med.

[12] Nakhoul IN, Kozuch P, Varma M (2006). Management of adult idiopathic thrombocytoia purpura. Clin Adv Hematol Oncol.

[13] Szmydki-Baran A, Adamowicz-Salach A, Gołębiowska- -Staroszczyk S (2008). Danazol – skuteczny lek drugiego rzutu w idiopatycznej plamicy małopłytkowej u dzieci. Opis 3 przypadków. Med Wieku Rozwoj.

[14] Kashiwagi H, Tomiyama Y (2013). Pathophysiology and management of primary immune thrombocytopenia. Int J Hematol.

[15] Ahn YS, Harrington WJ, Mylvaganam R, Ayub J, Pall LM (1985). Danazol therapy for autoimmune hemolytic anemia. Ann Intern Med.

[16] Fontana V, Dudkiewicz P, Ahn ER, Horstman L, Ahn YS (2011). Danazol therapy combined with intermittent application of chemotherapy induces lasting remission in myeloproliferative disorder (MPD). Hematology.

[17] Cervantes F, Hernandez-Boluda JC, Alvarez A, Nadal E, Montserrat E (2000). Danazol treatment of idiopathic myelofibrosis with severe anemia. Haematologica.

[18] Damaj G, Lefrere F, Canioni D (2002). Remission of transformed myelodysplastic syndrome with fibrosis after danazol therapy. Eur J Haematol.

[19] Tung S, Spaner DE (2012). A role for danazol in chronic lymphocytic leukemia. Leukemia.

[20] Darzynkiewicz Z, Bruno S, Del Bino G (1992). Features of apoptotic cells measured by flow cytometry. Cytometry.

[21] Wlodkowic D, Telford W, Skommer J, Darzynkiewicz Z (2011). Apoptosis and beyond: cytometry in studies of programmed cell death. Methods Cell Biol.

[22] Chou TC (2010). Drug combination studies and their synergy quantification using the Chou-Talalay method. Cancer Res.

[23] Matutes E, Polliack A (2000). Morphological and immunophenotypic features of chronic lymphocytic leukemia. Rev Clin Exp Hematol.

[24] Redaelli A, Laskin BL, Stephens JM, Botteman MF, Pashos CL (2004). The clinical and epidemiological burden of chronic lymphocytic leukemia. Eur J Cancer Care.

[25] Matalliotakis I, Neonaki M, Koumantaki Y, Goumenou A, Kyriakou D, Koumantakis E (2000). A randomized comparison of danazol and leuprolide acetate suppression of serum-soluble CD23 levels in endometriosis. Obstet Gynecol.

[26] Tomic J, Lichty B, Spaner DE (2011). Aberrant interferon-signaling is associated with aggressive CLL. Blood.

[27] Tanaka T (2009). Danazol regulates the functions of normal human endometrial stromal cell subpopulations by modifying endometrial cytokine networks. Int J Mol Med.

[28] Tanaka T, Umesaki N (2009). Danazol enhances Fas-mediated apoptosis in human endometrial epithelial cells within normal physiology. Int J Mol Med.

[29] Ueki K, Kumagai K, Yamashita H, Li ZL, Ueki M, Otsuki Y (2004). Expression of apoptosis-related proteins in adenomyotic uteri treated with danazol and GnRH agonists. Int J Gynecol Pathol.

[30] Kolb JP, Kern C, Quiney C, Roman V, Billard C (2003). Re-establishment of a normal apoptotic process as a therapeutic approach in B-CLL. Curr Drug Targets Cardiovasc Haematol Disord.

[31] Hallek M (2010). Therapy of chronic lymphocytic leukemia. Best Pract Res Clin Haematol.

[32] Morrison VA (2009). Infectious complications in patients with chronic lymphocytic leukemia: pathogenesis, spectrum of infection, and approaches to prophylaxis. Clin Lymphoma Myeloma.

[33] Robak T, Błonski JZ, Urbanska-Rys H, Błasińska-Morawiec M, Skotnicki AB (1999). 2-Chlorodeoxyadenosine (cladribine) in the treatment of patients with chronic lymphocytic leukemia 55 years old and younger. Leukemia.

[34] Furman RR (2010). Prognostic markers and stratification of chronic lymphocytic leukemia. Hematology Am Soc Hematol Educ Program.

[35] Shanafelt TD (2009). Predicting clinical outcome in CLL: how and why. Hematology Am Soc Hematol Educ Program.

[36] Zenz T, Fröhling S, Mertens D, Döhner H, Stilgenbauer S (2010). Moving from prognostic to predictive factors in chronic lymphocytic leukemia (CLL). Best Pract Res Clin Haematol.

